# Early Endothelial Signaling Transduction in Developing Lung Edema

**DOI:** 10.3390/life13061240

**Published:** 2023-05-24

**Authors:** Giuseppe Miserocchi

**Affiliations:** Department of Medicine and Surgery, Università di Milano Bicocca, 20900 Monza, Italy; giuseppe.miserocchi@unimib.it

**Keywords:** air–blood barrier, plasma membrane, lipid rafts, caveolae, hypoxia, fluid load

## Abstract

The lung promptly responds to edemagenic conditions through functional adaptations that contrast the increase in microvascular filtration. This review presents evidence for early signaling transduction by endothelial lung cells in two experimental animal models of edema, hypoxia exposure, and fluid overload (hydraulic edema). The potential role of specialized sites of the plasma membranes considered mobile signaling platforms, referred to as membrane rafts, that include caveolae and lipid rafts, is presented. The hypothesis is put forward that early changes in the lipid composition of the bilayer of the plasma membrane might trigger the signal transduction process when facing changes in the pericellular microenvironment caused by edema. Evidence is provided that for an increase in the extravascular lung water volume not exceeding 10%, changes in the composition of the plasma membrane of endothelial cells are evoked in response to mechanical stimuli from the interstitial compartment as well as chemical stimuli relating with changes in the concentration of the disassembled portions of structural macromolecules. In hypoxia, thinning of endothelial cells, a decrease in caveolae and AQP-1, and an increase in lipid rafts are observed. The interpretation of this response is that it favors oxygen diffusion and hinder trans-cellular water fluxes. In hydraulic edema, which generates greater capillary water leakages, an increase in cell volume and opposite changes in membrane rafts were observed; further, the remarkable increase in *caveolae* suggests a potential abluminal–luminal vesicular-dependent fluid reabsorption.

## 1. Introduction

Oxygen uptake in the lung is assured by an oversized architecture of the air–blood barrier that is essentially based on two geometrical features: a surface area approaching 100 m^2^ and a thickness as low as 0.5 µm [1]. Further, on morpho-functional ground, the macromolecular assembly of the interstitial compartment, separating the endothelial from the epithelial cell, enables the optimization of oxygen diffusion and, at the same time, prevents any fluid leakages from the blood capillaries [2]. These features reflect the macromolecular structure of the interstitial compartment that separates the endothelial from the epithelial cells. All inflammatory states, of either infectious (bacterial/viral) or sterile types (hypoxia exposure, surgery, and fluid overload), threaten the structural integrity of the interstitial macromolecular matrix by triggering the inflammatory cascade and, as a consequence, may cause fluid leakages from the capillary. The ensuing fluid accumulation in the interstitial compartment and in the alveoli, depending on the severity of the damage, hinders the process of oxygen uptake. This article is an up-to-date review focusing on the early role of the endothelial cellular signaling response occurring at the level of the air–blood barrier and aiming to shield the lung when facing the risk of developing edema. Two models of edema are considered: hypoxia exposure and fluid overload. The two models differ considering the damage induced on the macromolecular structure of the pulmonary interstitial compartment: hypoxia leads to an increase in water and solute permeability of the capillary barrier, while fluid overload mostly leads to a loss of the mechanical rigidity and compactness of the interstitial compartment. The two edema models actually share a common trait, namely the remarkable increase in the hydraulic pressure of the interstitial fluid resulting from the initial water leak from the capillaries. Accordingly, in both edema models, endothelial and epithelial cells are exposed to time-dependent mechanical stress. Evidence is provided for the difference in the signaling process eliciting a functional response aimed at buffering the specific initial perturbation in the lung water balance caused by either type of edema. Finally, when scaling up from cells to humans, functional considerations are put forward to link the signaling process with the inter-individual differences in vulnerability to lung edema. A hypothesis is put forward for potential inter-individual differences in the signaling process and/or inborn, or diseased-acquired, morpho-functional architecture of the air–blood unit. It is hoped that these data might help broaden the understanding of the early phase of perturbation in the lung fluid balance that is common to all inflammatory respiratory diseases.

## 2. The Air–Blood Barrier in Physiological Conditions

The terminal lung units include the “extra-alveolar space” and the “true alveolar space” [3], the latter only including the thin portion of the air–blood barrier (0.5 µm thick) devoted to gas exchange. At the level of the air–blood barrier, a dense package of molecules from the proteoglycan family (PG) guarantees impermeability to water and also provides mechanical stability to the lung parenchyma. PGs consist of a core protein with one or more covalently attached glycosaminoglycan (GAG) chains that are highly hydrophilic. The PG family comprises the large chondroitin sulphate sub-family (>1000 kDa) that provides stability to the collagen–elastin network component by filling the voids between these fibrillar molecules [4,5]. Further, the heparan sulphate PG sub-family (300–500 kDa) that extends to the intercellular clefts assures low microvascular permeability to water and proteins [3]. All PGs act as link proteins with other molecules, as well as with the cell surface through low-energy ionic and/or non-covalent bonds; the nature of these bonds allows mobility between structures and avoids shear stresses during lung movements. PGs represent a powerful shield against edema via specific physico-chemical features: (1) being highly hydrophilic, they can capture free water to form gel; (2) the increase in steric hindrance of the gel, coupled with the remarkable rigidity of the macromolecular structure, results in an increase in the hydraulic interstitial pressure from the physiological value −10 cmH_2_O [6] up to ~+5 cmH_2_O [7]. This increase remarkably offsets further microvascular filtration. At this stage, named interstitial lung edema, the increase in extravascular water is <10% [7]. A strict control on extravascular water is required as at the level of the air–blood barrier, there is scanty presence of lymphatics [8]. Fluid accumulation in the interstitial compartment would cause an increase in the intermolecular distance at PG’s binding sites; this, in turn, would decrease the intermolecular attraction forces according to the law of the square of the distance in the binding site. Moreover, sustained edemagenic conditions, proceeding along an inflammatory model, lead to the production of reactive oxygen species and activation of metalloproteases that ultimately cause remarkable fragmentation of PGs [9,10,11,12]. Fluid overload was generated by a slow rate of saline infusion (0.5 mL/(kg·min) [10], while hypoxic edema was obtained by exposure to 12% hypoxia for 3 h [12]. The two models differ in the sequence of matrix PG fragmentation in the air–blood barrier. Saline infusion mainly caused the fragmentation of large chondroitin sulphate PGs leading to an increase in tissue mechanical compliance [10]; conversely, in the hypoxia model, the main damage involved the heparan sulphate PGs leading to an increase in water and solute permeability [12]. When damaged PG molecules attain 60% control [12], fluid leakages increase remarkably, and severe edema develops with a time constant of a few minutes [13,14].

## 3. Early Cell Signaling in the Lung in Response to Developing Edema

**Figure 1:**
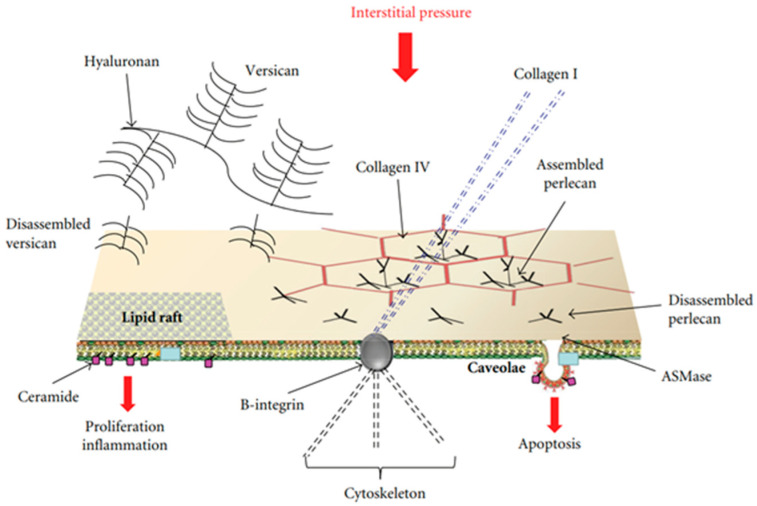
Possible model of lung cellular response to the increase in extravascular water not exceeding 10%. Membrane rafts (MRs), which include lipid rafts and caveolae, are shown. Cell signaling might be activated by mechanical stimulation caused by increased hydraulic pressure of the interstitial fluid through rigid links (collagen I, B-integrin, and cytoskeleton), and/or chemical activation of the MRs by fragments of matrix and basement membrane PGs (hyaluronan, perlecan, and versican). Data from [19].

Attention was focused on specialized sites of the plasma membranes that may be considered “mobile signaling platforms”, referred to as membrane rafts (MRs), that include lipid rafts and caveolae [15,16,17]. The hypothesis was developed that early cellular signaling might be reflected by changes in the plasma membrane’s composition of endothelial and epithelial cells at the level of the air–blood barrier in response to interstitial edema. MRs have a “quasi-crystalline” state and a different composition compared to the rest of the plasma membrane, in particular concerning lipid structure [16,17,18]. Figure 1 shows a possible model of the lung’s cellular response to developing edema [19].

**Figure 2:**
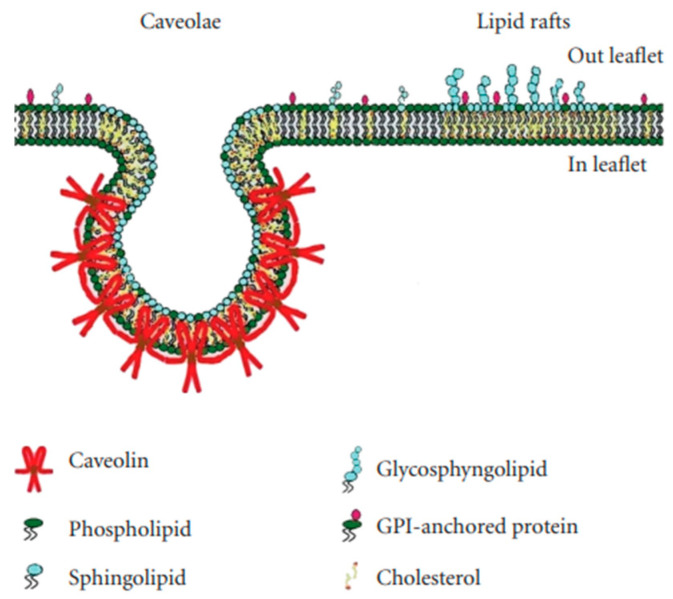
The structure of caveolae and lipid rafts. Data from [19].

Lipid rafts are essentially lipid-based domains. Caveolae are flask-shaped invaginations of the plasma membrane with a diameter of ~70 nm (Figure 2); they are protein-based domains due to the presence of caveolin [17,18].

Endothelial and epithelial cells are kept flat in a highly deformed state due to their strong attachments to the neighboring cells and the extracellular matrix. Accordingly, their “hard-wired” cytoskeleton might allow them to respond promptly to forces/pressures applied on their surface or changes in cellular volume based on the “tensegrity” concept [20]. Furthermore, the cytoskeleton can contribute to mechano-transduction by transmitting and modulating tension through rigid links involving B-integrin and collagen I as well as focal adhesion with adjacent cells and with the extracellular matrix [21]. MRs are highly movable, dynamic structures that further respond to changes in the concentration of disassembled portions of interstitial macromolecules including PGs (hyaluronan, versican, and perlecan) [22], an event occurring in a sustained edemagenic condition. Finally, the activation of these sites may be stimulated by the increase in water traffic. The hypothesis holds that changes in the lipid composition of the bilayer of the plasma membrane might contribute to triggering the signal transduction process involving MRs [23]. In both edema models, epithelial and endothelial cells are exposed to mechanical forces relating to the remarkable increase in hydraulic pressure of the interstitial fluid. In both models, data were acquired up to 3 h from the onset of the edemagenic condition.

## 4. Differences in Cellular Morphology Comparing the Two Edema Models: Hydraulic Type and Hypoxia Exposure

**Figure 3:**
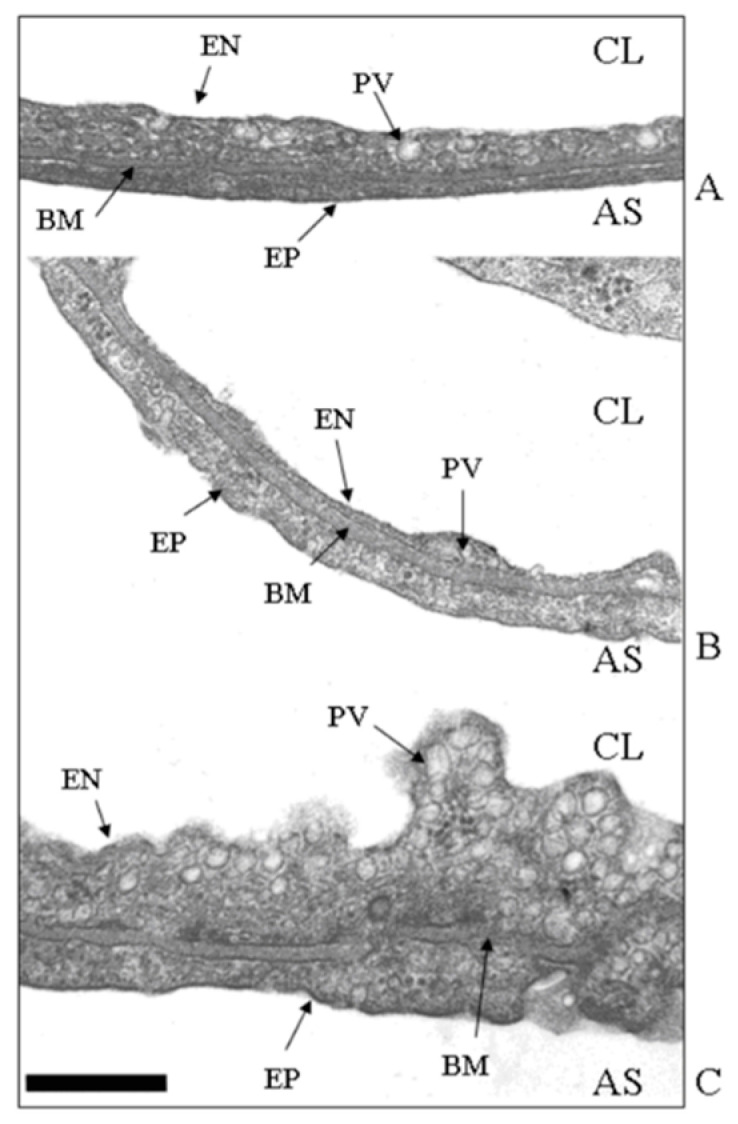
Ultrastructural appearance (TEM image, magnification ×66,000) of the thin portion of the air–blood barrier in control (**A**), after 3 h of hypoxia (**B**), and in hydraulic edema caused by 3 h of infusion of 0.5 mL/kg of saline solution. (**C**) CL, capillary lumen; AS, alveolar space; EN, endothelium; PV, plasmalemmal vesicle (caveolae); BM, basement membrane; EP, epithelium. Scale bar = 0.5 µm. Data from [24].

Figure 3A shows the thin portion of the air–blood barrier (the so–called “true alveolar space”), ~0.5 μm thick, simply made of endothelium (EN), epithelium (EP), and an intervening fused basement membrane (BM) representing the interstitial compartment; one plasmalemmal vesicle (PV, caveolae) is shown. Figure 3B shows that up until 3 h of hypoxia exposure, only an apparent thinning of the air–blood barrier occurred, with no increase in the number of caveolae. Conversely, Figure 3C shows that in the hydraulic type of edema caused by 3 h of saline infusion, the endothelial cell volume increased and the luminal side was highly irregular due to the increased number of cytosolic caveolae. In line with this finding, phospholipid phosphorous in the plasma membrane was found to increase, allowing an increase in its overall development [24]. Here, a specific involvement of caveolae in cases of perturbation in lung fluid balance is described; however, one should recall that caveolar expression is implicated in a variety of physiological and pathophysiological processes concerning the uptake/release of metabolites. Further, alterations or defects in caveolar function are associated with inflammatory states relating to immunological responses as well as the development of diseases such as cancer and cardiovascular and neurological disorders [25].

**Figure 4:**
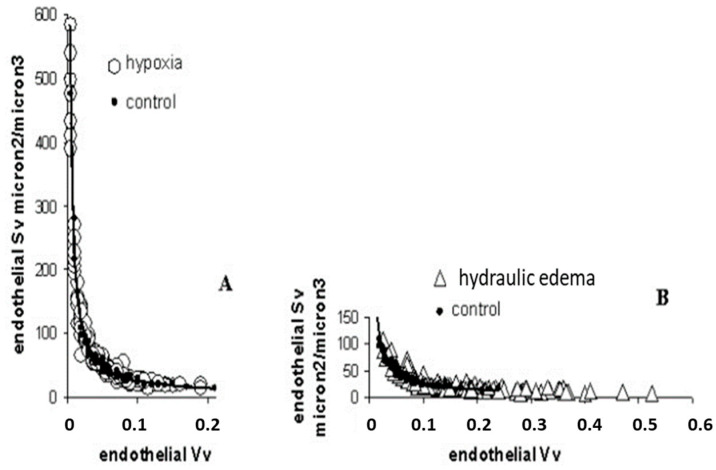
Surface and volume densities (Sv and Vv) for endothelial cells on hypoxia exposure (**A**) and hydraulic edema (**B**), relative to control. From [24].

Data from Figure 4A confirm the tendency of endothelial cells to decrease the cytoplasmic volume on hypoxia exposure becoming thinner, relative to the control; the opposite occurred in case of hydraulic edema (Figure 4B).

**Figure 5:**
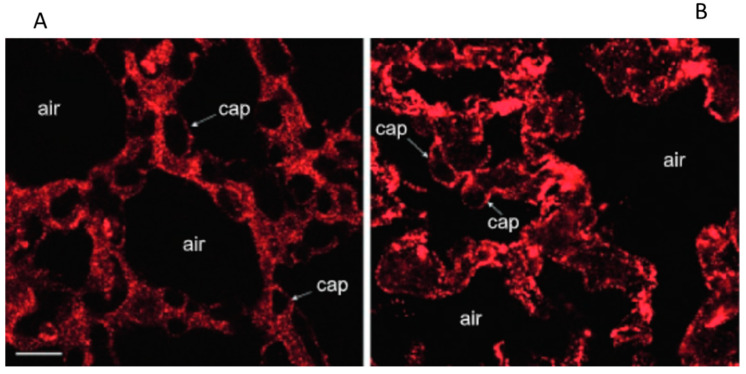
Confocal fluorescence images of Caveolin-1 immunostaining in frozen sections of lung parenchyma in hydraulic edema (**A**), relative to control (**B**); scale bar 12 µm. Data from rabbits [22].

In hydraulic edema confocal fluorescence images of Caveolin-1immunostaining (data from rabbit lungs) [22] confirmed its remarkable increase (Figure 5B), relative to the control (Figure 5A).

**Figure 6:**
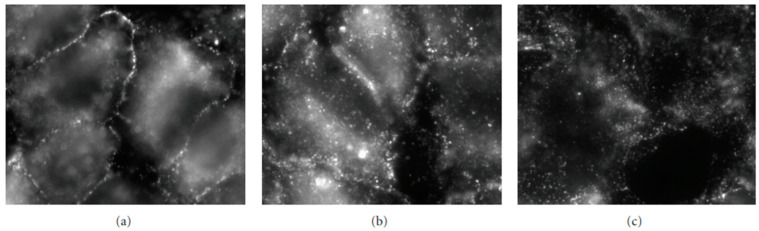
The figure shows the localization of Caveolin-1 in the plasma membrane in normoxic conditions (**a**) and its progressive translocation within the cytoplasm at 5 (**b**) and 24 h (**c**) of exposure to 5% hypoxia. Data from [26].

Figure 6 shows that, on hypoxia exposure, a progressive translocation of Caveolin-1 was observed from plasma membrane to cytoplasm with full intracellular localization at 24 h; this response is opposite to that occurring in the hydraulic edema [26].

**Figure 7:**
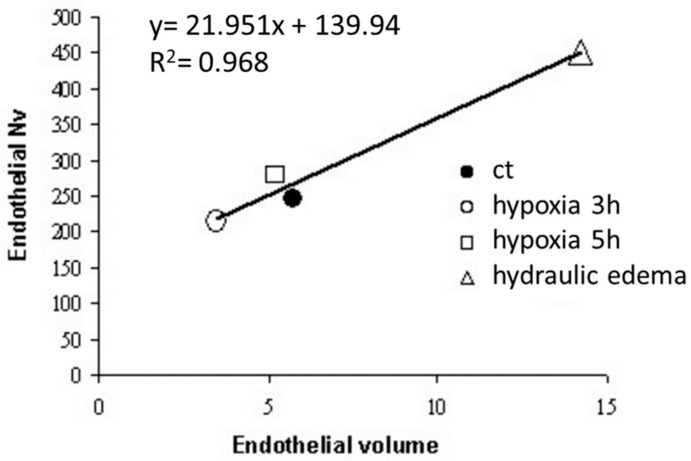
Regression between number of caveolae per unit volume of endothelial cells (Nv) plotted vs. volume of endothelial cells in control (ct), after 3 and 5 h of hypoxia and hydraulic edema (Data from [24].

Data from Figure 7 show a linear relationship between caveolar expression and volume of the endothelial cells; the expression of *caveolae* increased in hydraulic edema and decreased in hypoxia exposure.

**Figure 8:**
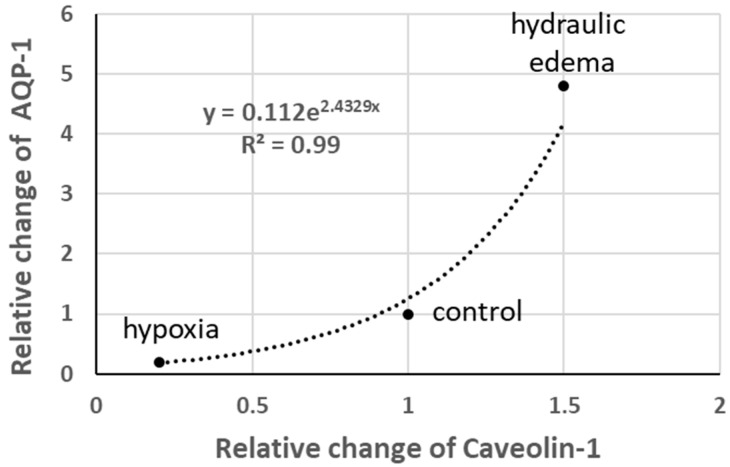
Relative changes in AQP-1 vs. relative changes in Caveolin-1 (data from [19]).

Aquaporins (AQP-1) are normally expressed in caveolae; interestingly, as shown in Figure 8, the relative change in AQP-1 follows a power function relative to the change in Caveolin-1. Note the five-fold increase in AQP-1 in hydraulic edema and its quasi-disappearance on hypoxia exposure.

Data from experimental models also indicate that the changes in the two forms of MRs are in opposite directions when comparing the two types of edema: caveolae increase and lipid rafts decrease in hydraulic edema, while the opposite occurs in hypoxia exposure [19,24]. This trend was confirmed by estimating the change in Caveolin-1 and of CD55 (marker of lipid rafts) in A549 alveolar human cells and in an alveolar cell line from human excised lungs (A30) exposed to hypoxia for up to 24 h [26]. The above data clearly indicate that, in developing interstitial edema, a significant re-modeling of the expression of MRs occurs, confirming that they behave as highly dynamic structures, potentially providing a prompt signaling platform responding to changes in the pericellular microenvironment.

A gene expression analysis was carried out in the hydraulic model of the edema. Cytokines TNFα and MT1 (a stress-response molecule) were up-regulated, representing a pro-inflammatory response; interestingly, IFNγ, a molecule leading to an increase in permeability and apoptosis, was down-regulated, a response clearly aimed at preserving the low permeability of the endothelial barrier. Further, MT1 genes (acting as antioxidant agents) markedly increased their expression, as well as bFGF [27]. In A549 and A30 cells exposed to 5% hypoxia for up to 24 h, a modest increase in mRNAs expression for CD55 and a slight decrease in Caveolin-1 were observed, which is in line with the morphological evidence [24].

## 5. Functional Comparison between the Hydraulic and the Hypoxic Model of Edema

An attempt can be made to relate the cellular response to the functional implications of the two types of edema at the organ level, considering the challenging data from Figure 8 in particular. The two models of edema investigated share the increase in interstitial pressure and a limited increase in extravascular water (<10% of control); however, one has no indications on the actual water flow among compartments. Recent extensive computational modeling of interstitial fluid in lung edema provided fluid velocity (Reynolds number averaging 0.006) and pressure profiles at capillary and interstitial levels, but no flows [28]. Flows could be estimated by multiplying the velocity by the surface through which the flow occurs. This estimate is difficult to compute; however, it is logical to admit that it is expected to vary in relation with the degree of recruitment of the capillary network. One can actually compare the two models of edema concerning this point. In the hydraulic type, left atrial pressure was almost doubled relative to the control [29], likely implying a recruitment of the capillary network and a possible increase in capillary pressure. These factors would cause a greater filtration rate compared to the hypoxia model where pre-capillary vasoconstriction would actually cause the de-recruitment of the capillary network.

The question is then: can one find a reason to justify the opposite signaling response when comparing the two edema models? A possible answer might consider what would be the most appropriate functional response to buffer the specific initial perturbation in lung water balance caused by either model of edema. In the case of the hydraulic type of edema, it is tempting to hypothesize that the increase in expression of caveolae might contribute to a polarized abluminal–luminal vesicular fluid reabsorption. This phenomenon would be favored by an increased expression of AQP-1, which is known to increase the water permeability of alveolar epithelial and endothelial cells [30]. So, in this kind of edema, the increase in water permeability at the endothelial level would favor the caveolar-dependent fluid drainage. The increase in volume of the endothelial cells involves complex K-Cl co-transport regulation and may well relate to changing plasma membrane stretching [31,32]. This interpretation fits with greater trans-vascular flows in hydraulic edema. It is not known how far the abluminal–luminal vesicular fluid reabsorption can balance the microvascular filtration; clearly, such an equilibrium becomes unbalanced toward edema when cellular integrity (both endothelial and epithelial) is disrupted.

**Figure 9:**
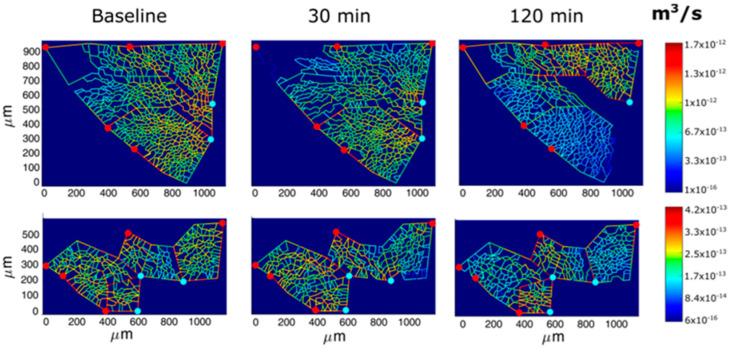
Computer-generated capillary perfusion pattern with a color-coded log-scale for two different alveolar–capillary networks (Network 1 and 2) in response to hypoxia. Red and light blue dots identify, respectively, arteriolar accesses and venular exits. The color panels show the capillary blood flow at different time points (baseline, 30 min, and 120 min of hypoxia exposure). Data from [33].

Following hypoxia exposure, the reduction in the endothelial cytoplasmic volume would decrease the overall thickness of the air–blood barrier and, moreover, silencing the expression of AQP-1 would limit trans-vascular fluid fluxes. These adaptive responses can obviously be regarded as aimed at favoring oxygen diffusion conductance. Furthermore, early signaling in hypoxia elicits precapillary vasoconstriction [33]. The functional significance of this reflex is to prevent the increase in hydraulic capillary pressure that represents, by and large, the more dangerous edemagenic factor [13]. Vasoconstriction implies the inhibition of potassium channels in smooth muscle cells, the activation of voltage-gated calcium channels, and the ensuing increase in cytosolic calcium [34]. Direct transpleural imaging in experimental animals allowed the time course of vasomotion in pulmonary microvessels (diameter < 100 nm) to be followed in response to 12% oxygen. Figure 9 shows a computer-generated capillary perfusion pattern with a color-coded log-scale for different alveolar–capillary network [33]. The vasoactive response was found to vary among lung regions; the blue color corresponds to the closure of microvessels in regions where an interstitial edema was preferentially developing. The figure also shows that color switching toward yellow corresponds to an increase in capillary flow, revealing blood flow re-direction from edematous toward intact lung regions [33].

A fractal geometry analysis showed that small asymmetries along the branching system might account for heterogeneity in both airway flow and blood flow distribution in terminal units [35]. The last paper also confirmed that regional lung perfusion reflects the complex interaction between vascular and surrounding alveolar pressures, particularly when local vasoconstriction occurs in response to hypoxia.

It has been reported that in edemagenic conditions, precapillary arterio-venous shunts are also activated upstream of the alveolar district [36,37,38]; this reflex response decreases capillary perfusion and therefore represents a further protection against the development of edemas.

Other factors are activated to signal hypoxia exposure. There are indications that oxidative stress is sensed in the lung by vagal unmyelinated afferent C-fibers originating from the so-called juxta-alveolar J-receptors, likely responding to the mechanical stimulation following the increase in tissue pressure in developing interstitial edemas [39]. Evidence has also been provided for the involvement of the mitochondrial electron transport chain as an oxygen sensor [40]. Further, mitochondria in smooth muscle cells of pulmonary artery act as oxygen sensors that transduce redox signals to regulate cellular ion channels and enzymes ultimately controlling hypoxic pulmonary vasoconstriction [41].

## 6. The Human Air–Blood Barrier Facing Edemagenic Conditions

Scaling up from cells to humans is a difficult, though necessary, step. It is well known that the adaptive response to edemagenic conditions impacts the control of lung extravascular water at the level of the air–blood barrier and, as a consequence, on the efficiency of the oxygen diffusion–transport function [42,43,44]. Oxygen uptake in the air–blood barrier reflects the complex interaction between oxygen membrane diffusion capacitance, blood-capacity (Hb), and capillary blood flow. The latter critically depends upon the degree of precapillary vasomotion and the increase in cardiac output, which are both elicited by hypoxia; accordingly, two more factors ought to be considered dealing with the oxygen equilibration along the capillary network: blood transit time and blood velocity [44]. The complex matching of all these factors reflects the efficiency of oxygen uptake in the lungs. Efficiency is known to vary remarkably among individuals, depending on the individual response to edemagenic conditions. A characteristic example is the exposure to hypoxia (high altitude), as one can partition HAPE-R (High Altitude Pulmonary Edema-Resistant) subjects from HAPE-S (High Altitude Pulmonary Edema-Sensitive) subjects [45]: the latter show a greater increase in pulmonary artery pressure, suggesting a stronger precapillary vasoconstriction as a defensive mechanism to decrease the perfusion of the capillary network and, at the same time, avoid an increase in capillary pressure [13,33,44].

In principle, the vulnerability to edemas and the loss of efficiency in gas exchanges may be related to the following: (1) the failure of the signaling process, and (2) individual adaptive response to the edemagenic condition relating to (3) deficient inborn or diseased-acquired morpho-functional structure of the air–blood unit. The first case seems unlikely to occur as precapillary vasoconstriction, the main shield against edema, has been reported to be much stronger in HAPE-S subjects [46].

Further, the adoption of the Multiple Inert Gas Exchange Technique (MIGET) [47] that enables the estimation of regional vasoconstriction in HAPE-S leads to a dissociation of perfusion with ventilation in developing edema: the unavoidable consequence was an incomplete equilibration of blood with alveolar O_2_ partial pressure. Regional lung perfusion reflects the complex interaction between vascular and peri-vascular pressure [33] as well as alveolar distending pressure, particularly when local vasoconstriction occurs in response to hypoxia [35]. Considering vasoconstriction as a defensive reflex shielding against edema formation, the finding of remarkable regional differences in vasoconstriction can be interpreted as the existence of potentially leaky sites along the microvascular district; accordingly, local signaling seems to be an efficient mechanism to protect against the local development of edemas.

## 7. Heterogeneity in Alveolar Morphology

Much interest was raised concerning point two and three above, aiming to detect individual structural/morphological features as well as inter-individual differences in the adaptive response to edemagenic conditions. Data in healthy humans showing a greater vulnerability to edema enabled a description of the specific morphological features of the terminal alveolar units. One such feature is the ratio of lung capillary blood volume (*Vc*) to the diffusion capacity of the alveolar membrane (*Dm*) [48]. These macro-parameters can be estimated from a common pneumological evaluation of lung diffusion properties. The functional significance of this ratio is that of comparing the overall surface of the capillary network (potentially providing microvascular filtration) with the extension of the overall surface area (available for gas diffusion). The distribution of the *Vc/Dm* ratio in the population studied was found to be normal, varying from ~1 to ~6 [44]. Based on computational modeling, the case of a high *Vc/Dm* is compatible with an alveoli of smaller diameter, implying a greater number of alveoli per unit lung volume [48]. The advantage of an alveoli of small radius (“the best possible acinus”) was also derived from numerical computational models as it would optimize oxygen diffusion [49,50]. One may add that a structural lung design based on small alveoli (the case of high *Vc/Dm*) offers a further advantage concerning the control of lung fluid balance. In fact, on geometrical ground, the overall capacitance of the interstitial peri-capillary compartment is larger compared to the case of high *Vc/Dm* [33]: the advantage is that in edemagenic conditions, more fluid can be hosted in the interstitial compartment preventing alveolar flooding. Subjects with a low *Vc/Dm* ratio had both an impairment of the oxygen diffusion and signs of perturbation in the lung fluid balance when exposed to highly edemagenic conditions (work in hypoxia, P_I_O_2_ 90 mmHg) [51]. Furthermore, in these subjects, the limitations of O_2_-diffusion led to a marked increase in cardiac output. The consequence of this cardiovascular response was the increase in blood velocity and the shortening of the blood transit time in the capillary district: the former increases the shear rate, which notably contributes to increasing microvascular permeability [52]; the latter prevents full equilibration of blood with alveolar PO_2_ [44].

Concerning point three above, a higher inborn microvascular permeability may also be hypothesized considering the morphological heterogeneity of the alveolar–capillary unit. Alveolar heterogeneity, which is likely to occur following any type of lung inflammation, alters the mechanical equilibrium between adjacent alveoli and, in turn, also the control of the extravascular fluid balance. Heterogeneity of alveolar size has been documented in experimental animals in physiological conditions [53,54] and found to reflect considerable differences in alveolar mechanical properties [54]. Yet, the clearance of alveolar edema can occur as long as the interstitial macromolecular assembly retains its mechanical integrity. A good example is alveolar fluid clearance in newborn rabbits at term [55]. Conversely, the reabsorption capacity is lost when the interstitial matrix is largely immature, in the case of premature rabbits [56], or largely disassembled [2]. Heterogeneous fragmentation of the molecular interstitial structure has been described in experimental lesional lung models [4,57]. Further, when heterogeneity ranges from alveolar atelectasis to over-distension, the inter-alveolar tissue stresses act to accelerate edema spreading on recruitment/de-recruitment of the respiratory units during the breathing cycle [58,59,60,61]. Large heterogeneity in alveolar size also occurs in human emphysema, a pathology characteristically exposed to the risk of edema [62].

## 8. Conclusions and Summary of Unknown Points to Be Addressed in Future Studies

Studying the early phase of edemagenic conditions allowed us to identify the innate response of the lung to counteract the perturbation of water balance. The indications are that the response is triggered via mechanosensing evoked by the increase in interstitial pressure following the initial increase in capillary leak. This conclusion is in line with the concept of coupling “*time-dependent mechanosensory with context-specific adaptive responses to rebalance the cell homeostatic state*” [63]. The message from these studies is that as long as the integrity of the macromolecular structure is preserved, the increase in extravascular water is kept within 10%. A further indication is that the lung can resist for a long time in this condition.

A point to be addressed is how to ”merge’ these results with human’’ clinical data on severe forms of lung edema as well as with data from experimental models of lung lesions. The first important observation is that routine clinical assessments of patients are unable to detect the early phase of increased extravascular fluid in the lungs [64]. This point is crucial considering that the time constant for developing severe edema is a matter of minutes [13,14]. Valid help would come from an in vivo estimate of vascular permeability [65]. It is of interest to recall the recently proposed methods allowing to relate the time course of edema based on water evaporation from exhaled air [66].

The reported clinical heterogeneity of ARDS [67,68] raised the point of inter-individual differences in lung vulnerability concerning edema. This concept is actually supported by the finding of a correlation between individual morpho-functional features of the terminal alveolar units and the degree of lung water perturbation when facing edemagenic conditions [44,48].

Concerning the recovery from severe lung lesions, further points have been addressed.

In the case of sepsis-induced lung injuries, novel regulators of the inflammatory response have been proposed [69]. In models of lung ischemia/reperfusion injuries, the point of alleviating endoplasmic reticulum stress in alveolar epithelial type II cells has been considered [70].

Considering high-altitude pulmonary edema, it was possible to identify a distinct, specific subset of monocyte linked with down-regulation of HIF-1α that might be implicated in the development of the disease [71]. Further, in response to hypoxia, non-classical monocytes in the lung have been identified that can sense hypoxia and promote vascular remodeling, leading to the development of pulmonary hypertension [72].

At the therapeutic level, an interesting report focuses on the role of ropivacaine to inhibit/prevent pressure-induced lung endothelial hyperpermeability [73]. Further, soy isoflavone has been proposed to reduce pulmonary edema in a dose-dependent manner [74]. Mesenchymal stem cell therapies are also reported as promising therapeutic agents in ARDS [75].

Considering the transition from the early postlesional inflammatory stage to the subsiding phase leading to and remodeling of the lung structure, the role of ulinastatin was shown to promote the process of efferocytosis, namely the clearing process of apoptotic neutrophils operated by macrophages to favor repair [76].

Finally, remodeling of the extracellular matrix has been considered a long-range effect of increased microvascular permeability leading to post-injury lung fibrosis [77,78].

One can speculate about the fact that pulmonary hypertension progressively develops as a consequence of pulmonary fibrosis [79]. One could hypothesize that the functional response to high microvascular permeability (either inborn or acquired) might stimulate fibrosis deposition on the precapillary side, aiming to protect the capillary network from over-perfusion and an increase in hydraulic pressure. Accordingly, the progressive limitation of capillary blood flow could represent a long-term adaptive response shielding against the risk of lung edema. This interpretation is in keeping with the finding of a decrease in the density of microvessels in idiopathic fibrosis [80]. The counterpart of this defensive adaptive response is that fibrosis is due to become a case of maladaptive compensation as it leads, over time, to an increase in right-ventricular afterload. The pathogenesis of pulmonary fibrosis is currently the object of growing interest to prevent the progression of the disease. The role of trans-differentiation of lung fibroblasts into myofibroblasts proliferation and the ensuing extensive deposition of extracellular matrix are key challenging points from a therapeutic point of view [81,82,83].
